# Amplicon sequencing of anaerobic digester microbial communities following *Caldicellulosiruptor bescii* pretreatment of wastewater substrates

**DOI:** 10.1128/mra.00104-26

**Published:** 2026-03-23

**Authors:** Maliea A. Nipko, Zachary T. Aanderud

**Affiliations:** 1 Department of Microbiology and Molecular Biology, Brigham Young University 6756, Provo, Utah, USA; 2 Department of Plant and Wildlife Sciences, Brigham Young University 6756, Provo, Utah, USA; Rochester Institute of Technology, Rochester, New York, USA

**Keywords:** *Caldicellulosiruptor bescii*, amplicon, waste activated sewage, V4 16S, anaerobic digestion

## Abstract

We report 16S rDNA sequencing of anaerobic digester microbiomes processing primary clarifier sludge, anaerobic digestate, and waste activated sewage with and without biological pretreatment of *Caldicellulosiruptor bescii*. DNA was extracted from pellet-associated and free-living microhabitats. Amplicon sequencing with Illumina MiSeq was performed targeting the V4 region of 16S rDNA.

## ANNOUNCEMENT

Anaerobic digestion (AD) is a biological process where microorganisms convert organic waste to biogas (1–3). AD efficiency is dependent on the accessibility of organic matter, such as lignocellulose, as well as the composition of microbial consortium (4–6). Pretreatment may increase the bioavailability of recalcitrant substances in AD substrates (7). *Caldicellulosiruptor bescii* has emerged as an efficient biological pretreatment due to its capacity to solubilize lignin while simultaneously metabolizing monosaccharides (8, 9). The primary metabolic product of *C. bescii* is acetate, which may be further metabolized by acetoclastic methanogens (9). However, the impact of *C. bescii* on various substrates and AD communities has yet to be determined.

We implemented a biological pretreatment on primary clarifier sludge, post-anaerobic digestion effluent, and waste activated sludge in a two-stage system. Substrates were pretreated with *C. bescii* for 4 days followed by 12 days of mesophilic AD. Samples were centrifuged at 5,000 rpm for 5 min to separate the supernatant and pellet, designated as free-living or pellet-associated microbial community, respectively.

DNA was extracted from 800 μL of supernatant and pellets of each sample using QIAamp PowerFecal Pro DNA kit (Qiagen, Germany) following the manufacturer’s protocol. DNA quantity and quality were assessed spectrophotometrically on a NanoDrop One Microvolume UV-Vis Spectrophotometer (Thermo Fisher Scientific, Waltham, MA USA). A two-step PCR amplification of the V4 region was conducted using the Platinum II Hot-Start PCR Master Mixes (Invitrogen, Massachusetts) and primers 515F (5′-GTGYCAGCMGCCGCGGTAA-3′) and 806R (5′-GGACTACNVGGGTWTCTAAT-3′). The first round of PCR conditions included initial denaturation for 3 min at 94°C followed by 35 cycles of 45 s at 94^o^C, 60 s at 50^o^C, and 60 s at 72°C. Final elongation was run for 10 min at 72^o^C. Each sample was run in triplicate for the first round of PCR, after which the triplicates were combined and diluted 50-fold for a second round of PCR with indexing primers. The second round of PCR included initial denaturing at 94^o^C for 1 min followed by 12 cycles of 15 s at 94^o^C, 15 s at 64^o^C, and 60 s at 72^o^C. Final elongation followed at 72^o^C for 3 min. Dual indexed libraries were purified with AMPure XP Beads for DNA Cleanup (Beckman Coulter, California). Libraries were sequenced on the Illumina MiSeq using 600-cycle v3 chemistry (2 × 300 paired-end sequencing, Illumina, California).

**Fig 1:**
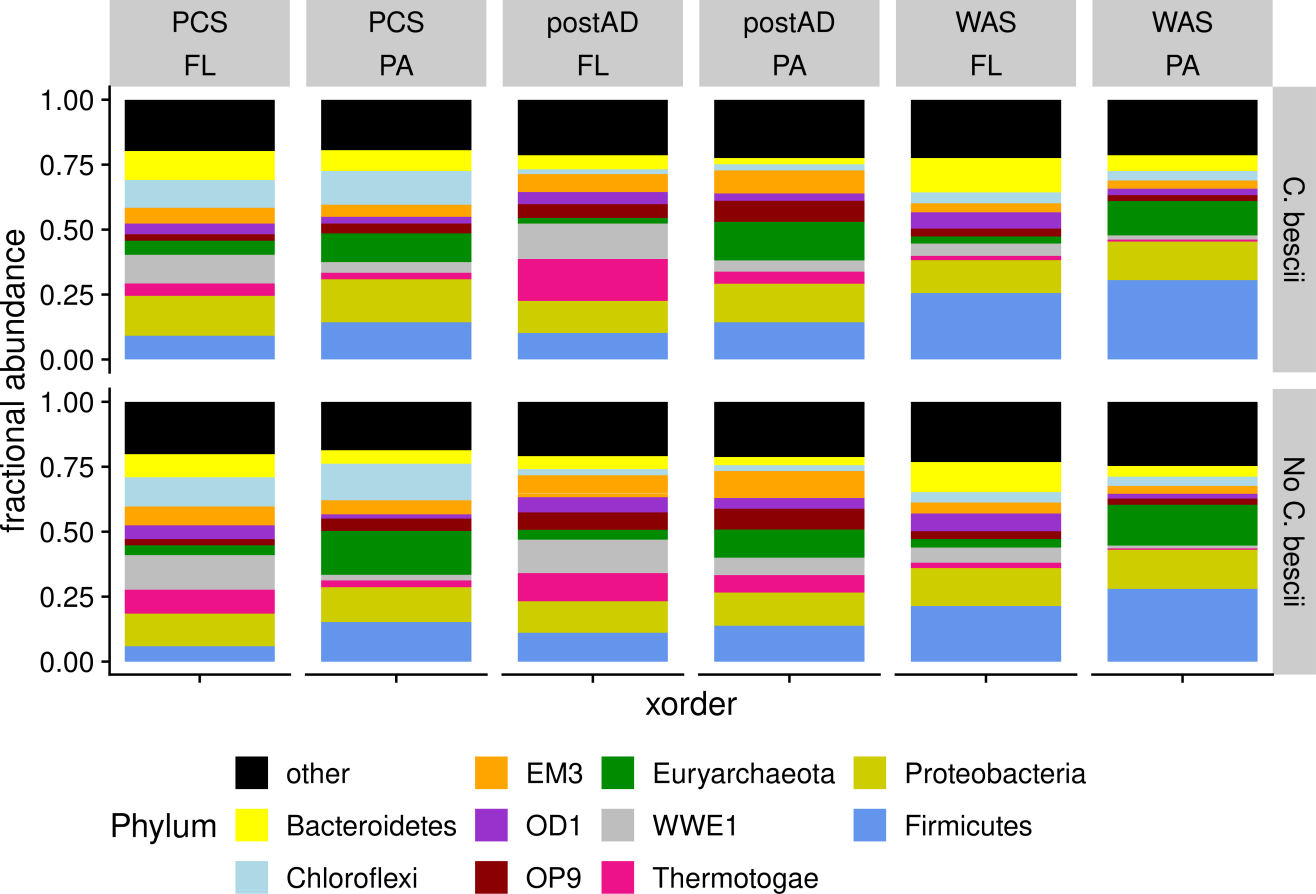
The microbiota for anaerobic digesters fed primary clarifier sludge (PCS), post anaerobic digestion effluent (postAD), and waste activated sludge (WAS) stratified by pretreatment with *C. bescii*. The microbial composition is depicted by phyla, rarefied to 14,988 reads per sample. ASVs greater than 4% are shown individually.

Quality control, assembly, and taxonomic classification were performed with the QIIME 2 2021.2 (10) bioinformatic pipeline. The samples were demultiplexed and denoised using DADA2 (11). After denoising and chimera removal, 2,446,006 reads across 64 samples and 3,753 features remained. The samples were rarefied (subsampled without replacement) to 14,988 sequences per sample. Samples, classified with the SILVA database, were dominated by the phyla *Firmicutes*, *Proteobacteria*, *Euryarchaeota*, and *Thermotogae* (Fig. 1). Microbial communities of the AD were dependent on the substrate, pretreatment process, and microhabitat (PERMANOVA substrate, *F* = 28.0, *P* < 0.005, df = 2; microhabitat, *F* = 20.9, *P* < 0.005, df = 1; pretreatment, *F* = 2.67, *P* < 0.05, df = 1) (Table 1).

**TABLE 1:** Sample ID table distinguishing read efficiency, treatment, and substrate^*a*^

Sample ID	SRA sample name	SRA number	Total reads	Filtered reads	Total reads that passed the filter (%)	Pretreatment	Microhabitat	Substrate
MAH003	AD sample 1	SRR32597729	57,224	50,011	87.4	*C. bescii*	PA	postAD
MAH004	AD sample 2	SRR32597728	55,091	47,699	86.58	*C. bescii*	FL	postAD
MAH007	AD sample 3	SRR32597717	52,035	44,746	85.99	No *C. bescii*	PA	postAD
MAH008	AD sample 4	SRR32597706	54,269	46,885	86.39	No *C. bescii*	FL	postAD
MAH011	AD sample 5	SRR32597695	40,879	35,334	86.44	*C. bescii*	PA	postAD
MAH012	AD sample 6	SRR32597684	51,718	45,185	87.37	*C. bescii*	FL	postAD
MAH015	AD sample 7	SRR32597673	50,996	44,124	86.52	No *C. bescii*	PA	postAD
MAH016	AD sample 8	SRR32597668	48,151	42,146	87.53	No *C. bescii*	FL	postAD
MAH019	AD sample 9	SRR32597667	57,753	49,764	*86.17*	*C. bescii*	PA	postAD
MAH020	AD sample 10	SRR32597666	50,826	44,148	86.86	*C. bescii*	FL	postAD
MAH023	AD sample 11	SRR32597726	56,800	48,921	86.13	No *C. bescii*	PA	postAD
MAH024	AD sample 12	SRR32597726	56,658	49,254	86.93	No *C. bescii*	FL	postAD
MAH027	AD sample 13	SRR32597725	50,324	43,546	*86.53*	*C. bescii*	PA	postAD
MAH028	AD sample 14	SRR32597724	43,954	38,275	87.08	*C. bescii*	FL	postAD
MAH031	AD sample 15	SRR32597723	44,668	38,398	85.96	No *C. bescii*	PA	postAD
MAH032	AD sample 16	SRR32597722	63,340	55,718	87.97	No *C. bescii*	FL	postAD
MAH035	AD sample 17	SRR32597721	45,100	38,674	85.75	*C. bescii*	PA	postAD
MAH036	AD sample 18	SRR32597720	46,312	39,904	86.16	*C. bescii*	FL	postAD
MAH039	AD sample 19	SRR32597719	44,372	38,424	86.6	No *C. bescii*	PA	postAD
MAH040	AD sample 20	SRR32597718	45,536	39,837	87.48	No *C. bescii*	FL	postAD
MAH043	AD sample 21	SRR32597716	40,759	35,233	86.44	*C. bescii*	PA	postAD
MAH044	AD sample 22	SRR32597715	41,321	36,746	88.93	*C. bescii*	FL	postAD
MAH047	AD sample 23	SRR32597714	46,484	40,740	87.64	No *C. bescii*	PA	postAD
MAH048	AD sample 24	SRR32597713	47,197	40,894	86.65	No *C. bescii*	FL	postAD
MAH051	AD sample 25	SRR32597712	48,236	42,230	87.55	*C. bescii*	PA	PCS
MAH052	AD sample 26	SRR32597711	44,227	38,375	86.77	*C. bescii*	FL	PCS
MAH053	AD sample 27	SRR32597710	45,890	40,395	88.03	No *C. bescii*	PA	PCS
MAH054	AD sample 28	SRR32597709	37,594	32,585	86.68	No *C. bescii*	FL	PCS
MAH057	AD sample 29	SRR32597708	47,662	41,476	87.02	*C. bescii*	PA	PCS
MAH058	AD sample 30	SRR32597707	46,093	40,134	87.07	*C. bescii*	FL	PCS
MAH061	AD sample 31	SRR32597705	59,250	51,536	86.98	No *C. bescii*	PA	PCS
MAH062	AD sample 32	SRR32597704	54,103	46,994	86.86	No *C. bescii*	FL	PCS
MAH065	AD sample 33	SRR32597703	43,218	37,840	87.56	*C. bescii*	PA	PCS
MAH066	AD sample 34	SRR32597702	34,821	30,347	87.15	*C. bescii*	FL	PCS
MAH069	AD sample 35	SRR32597701	36,819	31,848	86.5	No *C. bescii*	PA	PCS
MAH070	AD sample 36	SRR32597700	37,014	32,150	86.86	No *C. bescii*	FL	PCS
MAH073	AD sample 37	SRR32597699	51,475	44,540	86.53	*C. bescii*	PA	PCS
MAH074	AD sample 38	SRR32597698	34,558	29,885	86.48	*C. bescii*	FL	PCS
MAH077	AD sample 39	SRR32597697	39,239	33,924	86.45	No *C. bescii*	PA	PCS
MAH078	AD sample 40	SRR32597696	36,267	32,233	88.88	No *C. bescii*	FL	PCS
MAH083	AD sample 41	SRR32597694	38,555	33,324	86.43	No *C. bescii*	PA	PCS
MAH084	AD sample 42	SRR32597693	39,530	34,038	86.11	No *C. bescii*	FL	PCS
MAH087	AD sample 43	SRR32597692	51,613	44,882	86.96	*C. bescii*	PA	PCS
MAH088	AD sample 44	SRR32597691	41,462	36,405	87.8	*C. bescii*	FL	PCS
MAH091	AD sample 45	SRR32597690	41,289	35,464	85.89	No *C. bescii*	PA	PCS
MAH092	AD sample 46	SRR32597689	38,697	34,465	89.06	No *C. bescii*	FL	PCS
MAH145	AD sample 47	SRR32597688	63,889	55,255	86.49	No *C. bescii*	PA	WAS
MAH146	AD sample 48	SRR32597687	46,977	40,940	87.15	No *C. bescii*	FL	WAS
MAH151	AD sample 49	SRR32597686	49,652	42,790	86.18	No *C. bescii*	PA	WAS
MAH152	AD sample 50	SRR32597685	20,311	17,604	86.67	No *C. bescii*	FL	WAS
MAH153	AD sample 51	SRR32597683	42,676	37,190	87.14	*C. bescii*	PA	WAS
MAH154	AD sample 52	SRR32597682	35,256	30,900	87.64	*C. bescii*	FL	WAS
MAH159	AD sample 53	SRR32597681	40,141	34,735	86.53	*C. bescii*	PA	WAS
MAH160	AD sample 54	SRR32597680	38,199	33,673	88.15	*C. bescii*	FL	WAS
MAH165	AD sample 55	SRR32597679	52,997	46,586	87.9	*C. bescii*	PA	WAS
MAH166	AD sample 56	SRR32597678	45,945	40,213	87.52	*C. bescii*	FL	WAS
MAH169	AD sample 57	SRR32597677	60,018	52,127	86.85	*C. bescii*	PA	WAS
MAH170	AD sample 58	SRR32597676	66,998	58,790	87.75	*C. bescii*	FL	WAS
MAH171	AD sample 59	SRR32597675	67,413	58,958	87.46	No *C. bescii*	PA	WAS
MAH172	AD sample 60	SRR32597674	57,939	51,229	88.42	No *C. bescii*	FL	WAS
MAH175	AD sample 61	SRR32597672	67,317	58,637	87.11	No *C. bescii*	PA	WAS
MAH176	AD sample 62	SRR32597671	71,637	63,849	89.13	No *C. bescii*	FL	WAS
MAH179	AD sample 63	SRR32597670	44,770	39,133	87.41	No *C. bescii*	PA	WAS
MAH180	AD sample 64	SRR32597669	38,642	33,797	87.46	No *C. bescii*	FL	WAS

^*a*^ FL, free-living; PA, pellet-associated; PCS, primary clarifier sludge.

## Data Availability

The 16S rDNA sequences are deposited in the NCBI Sequence Read Archive (SRA) repository under BioProject ID PRJNA1232727; SRA sequences are listed in Table 1. The version described in this paper is the first version.
